# Adaptations of early development to local spawning temperature in anadromous populations of pike (*Esox lucius*)

**DOI:** 10.1186/s12862-019-1475-3

**Published:** 2019-07-22

**Authors:** Johanna Sunde, Per Larsson, Anders Forsman

**Affiliations:** Ecology and Evolution in Microbial Model Systems, EEMiS, Department of Biology and Environmental Science, Linnaeus University, SE-392 31 Kalmar, Sweden

**Keywords:** Adaptation, Climate change, *Esox lucius*, Pike, Temperature

## Abstract

**Background:**

In the wake of climate change many environments will be exposed to increased and more variable temperatures. Knowledge about how species and populations respond to altered temperature regimes is therefore important to improve projections of how ecosystems will be affected by global warming, and to aid management. We conducted a common garden, split-brood temperature gradient (4.5 °C, 9.7 °C and 12.3 °C) experiment to study the effects of temperature in two populations (10 families from each population) of anadromous pike (*Esox lucius*) that normally experience different temperatures during spawning. Four offspring performance measures (hatching success, day degrees until hatching, fry survival, and fry body length) were compared between populations and among families.

**Results:**

Temperature affected all performance measures in a population-specific manner. Low temperature had a positive effect on the Harfjärden population and a negative effect on the Lervik population. Further, the effects of temperature differed among families within populations.

**Conclusions:**

The population-specific responses to temperature indicate genetic differentiation in developmental plasticity between populations, and may reflect an adaptation to low temperature during early fry development in Harfjärden, where the stream leading up to the wetland dries out relatively early in the spring, forcing individuals to spawn early. The family-specific responses to temperature treatment indicate presence of genetic variation for developmental plasticity (G x E) within both populations. Protecting between- and within-population genetic variation for developmental plasticity and high temperature-related adaptive potential of early life history traits will be key to long-term viability and persistence in the face of continued climate change.

**Electronic supplementary material:**

The online version of this article (10.1186/s12862-019-1475-3) contains supplementary material, which is available to authorized users.

## Background

Environmental conditions (e.g. hydrogeography, temperature and salinity) in natural habitats are changing worldwide due to climate change [1, 2], and global warming is one of the most important anthropogenic disturbances for nature [3, 4]. There is a general agreement that average temperatures will continue to increase, although the rate and magnitude is predicted to vary geographically. Besides the overall elevation in mean temperature, fluctuations in temperature are expected to increase both within and among years [1]. How different species and populations will be affected and how they will be able to cope with altered conditions depends in part on their ecology, genetic architecture, capacity for developmental plasticity, phenotypic flexibility, and on other potential environmental constraints [5–12].

Temperature affects physiological processes which are essential for organisms, and is therefore an important environmental factor that influences the wellbeing, and ultimately survival, of organisms [13, 14]. To cope with spatiotemporal variations in temperature, species utilize different thermoregulatory strategies. Endotherms can use heat generated via internal physiological processes to regulate their body temperature [15]. Ectotherms that are not able to produce their own heat, instead rely on external heat from the surrounding environment and on behavioral thermoregulation (e.g. moving between colder and warmer environments, and by sun basking) to regulate their internal temperature [16–20]. This potentially makes ectotherms especially vulnerable to temperature changes [21].

In aquatic ecosystems, most species including fishes are ectotherms. Changes in water temperature owing to climate change might therefore have vast effects on the wellbeing of such ecosystems. One example of an ectothermic fish species is pike (*Esox lucius*). Pike is a large, long-lived species with a circumpolar distribution on the northern hemisphere. As a top-predator it regulates lower trophic levels by top-down control [22–24]. It is also a valued species for commercial and recreational fishing [25, 26] and an important model species in studies of ecology and evolution [27].

In the Baltic Sea, two different spawning ecotypes of pike, anadromous and resident, co-occur [28], and inhabit the coastal areas. The two spawning ecotypes are sympatric in the Baltic Sea for the main part of the year, and separate only for a short period during spawning, when the anadromous individuals migrate to their natal freshwater habitats (i.e. rivers and wetlands), whilst the resident individuals spawn in the brackish waters along the coast [22, 29–31]. The adult anadromous pike generally leave the wetlands shortly after spawning to return to the sea to forage [30, 32, 33], and the juveniles commonly stay less than 1 month before migrating to the Baltic Sea [31]. The homing behaviour of anadromous pike [34–36] has allowed anadromous sub-populations using closely located spawning areas to become genetically [22, 37] and phenotypically [38–41] differentiated.

Previous studies have found that pike populations in the Baltic Sea harbor local adaptations in several morphological and life-history traits, such as hatching success and larval survival [39], growth rate and body size [41], vertebral count [38], and salinity tolerance [40]. However, it is not known whether pike populations differ in temperature tolerance, or whether standing genetic variation, variation for developmental plasticity, or phenotypic flexibility make them capable of coping with changes in temperature at the rates associated with ongoing and future climate change.

**Fig. 1:**
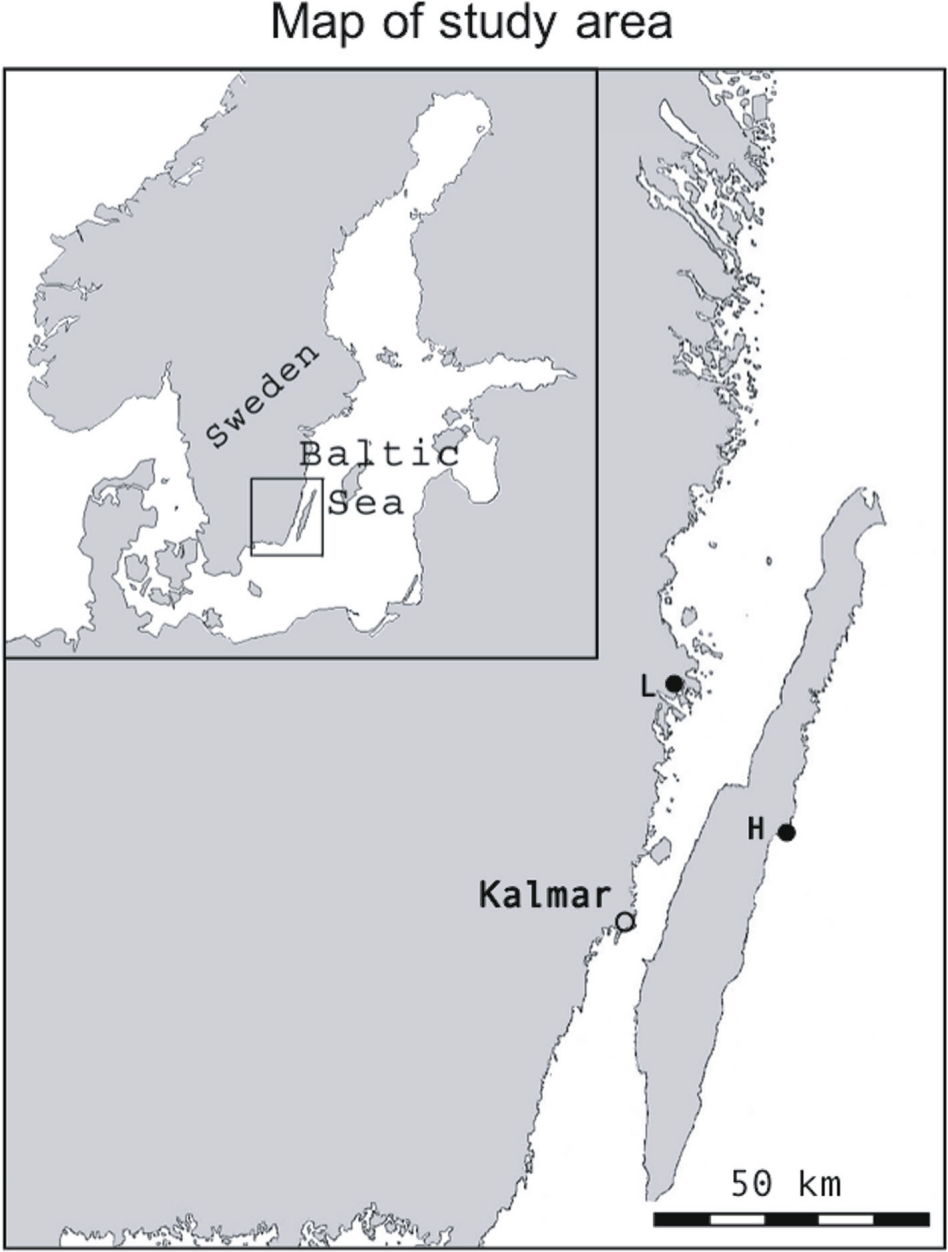
Map of the study area in the Southeast of Sweden. The map is showing the spawning locations for the two anadromous *Esox lucius* populations (H: Harfjärden, L: Lervik) included in the experiment. The map was generated in Adobe Photoshop CC, version 2015.0.1 by modifying two base maps (one of Scandinavia and one of Sweden), which are available under non-restrictive creative commons license from Wikimedia Commons, https://commons.wikimedia.org/wiki/File:Scandinavia-template.png and https://commons.wikimedia.org/wiki/File:Sweden_location_map.svg

The objective of the present study was to compare temperature tolerance of two anadromous subpopulations of pike from the Baltic Sea (Fig. 1) that differ in spawning time and in temperature regimes experienced during spawning and incubation of eggs and embryos. The phenological difference in the timing of spawning between the populations is likely caused by the waterflow in the stream leading up to one of the wetlands declining fast after the spring flood (County Administrative Board Kalmar; waterflow estimates based on data from the Swedish Meteorological and Hydrological Institute, SMHI [42]). The decline in water results in that the pikes are only able to enter the wetland for a restricted period when the waterflow is higher, and the stream then dries out early in the spring. Because of this time constraint the spawning and egg development occur in colder water (also supported by results reported in the present study). It can thus be hypothesized that adaptations of early development to local temperatures have evolved, such that the effects of temperature are population specific. To compare temperature tolerance of early life history between the two populations and to investigate and compare genetic variation for temperature related developmental plasticity of offspring performances, a split-brood experiment in a temperature gradient (4.5 °C, 9.7 °C and 12.3 °C) was carried out. Effects on hatching success, day degrees until hatching, fry survival, and fry body length were compared among temperatures, between populations, and among families.

## Results

The overall findings of this study were: *i*) spawning time and spawning temperature observed in the field differed between the populations; *ii*) temperature affected all four offspring performance measures; *iii*) effects of temperature differed between the two populations, indicating genetic differentiation between the populations in developmental plasticity of early life history traits; and *iv*) effects of temperature differed among families within both populations (G x E), indicating the presence of genetic variation for developmental plasticity also within populations.

### Field observations and temperature measurements

**Fig. 2:**
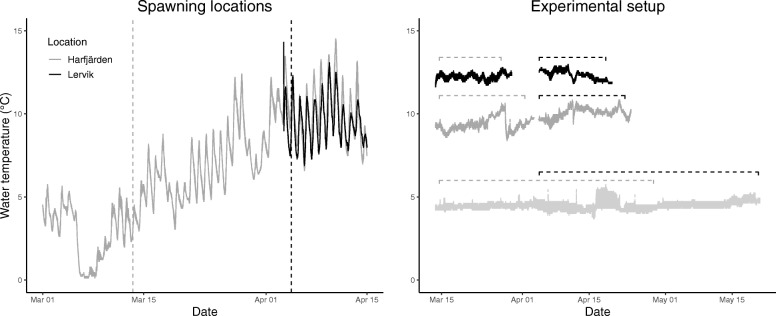
Water temperature in the wild and in the experimental setup in the lab. *Left plot:* Solid lines show the water temperatures in the natural spawning locations (measured every 30 min), and the dashed lines indicate when the two populations started spawning (i.e. when the experiment was started). Grey lines represent Harfjärden, and black lines represent Lervik. *Right plot:* Solid lines show the water temperatures in the three treatments in the experimental setup (black: high temperature treatment (12.3 °C), dark grey: medium temperature treatment (9.7 °C), and light grey: low temperature treatment (4.5 °C)). Horizontal dashed lines indicate the duration of the experiment for the two populations (grey line represents Harfjärden, and black line represents Lervik)

Spawning time started 3 weeks earlier in Harfjärden (March 14) than in Lervik (April 4). Temperature at the initiation of spawning was lower in Harfjärden than in Lervik (4.3 ± 1.1 °C and 9.4 ± 1.6 °C respectively, mean ± s.d., see Fig. 2a).

Data from the temperature loggers in the laboratory showed that the average temperature for the treatments was 4.5 ± 0.3 °C, 9.7 ± 0.5 °C, and 12.3 ± 0.3 (mean ± s.d.) in the low, medium and high temperature treatment respectively. Despite that the experiment had to be split into two periods (one for each population), the temperature within treatments were similar for both populations (low: 4.4 ± 0.3 °C and 4.5 ± 0.3 °C; medium: 9.4 ± 0.41 °C and 10.1 ± 0.3 °C; and high: 12.2 ± 0.2 °C and 12.4 ± 0.27 °C, mean ± s.d. for Harfjärden and Lervik respectively, see Fig. 2b).

### Effects of rearing temperature on overall offspring performance

The effect of rearing temperature on overall offspring performance (hatching success, day degrees until hatching, fry survival, and fry body length) differed between the populations (MANOVA, effect of population by temperature treatment interaction, Wilks Lambda, Λ = 0.90, *P* = 0.017; effect of population: Λ = 0.63, *P* = < 0.0001; effect of temperature treatment: Λ = 0.059, *P* = < 0.0001). Results from separate analyses for each of the four performance measures are reported below.

### Hatching success

**Fig. 3:**
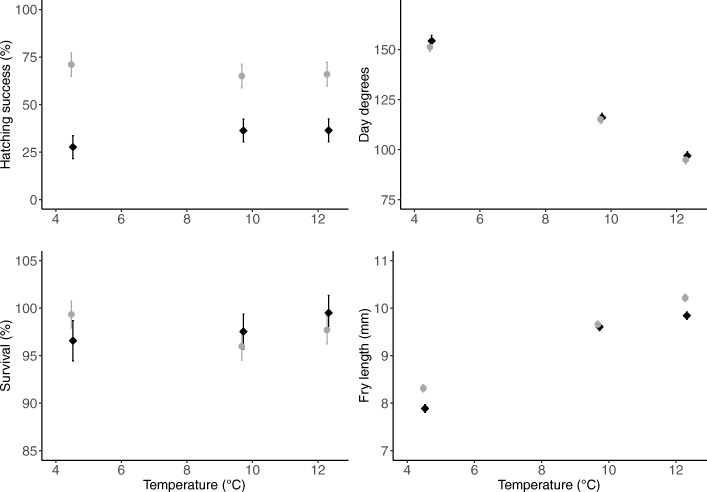
Effects of temperature on offspring performance. Effects of temperature on four offspring performance measures (hatching success of eggs, day degrees until hatching, survival during the first 5 days post hatching, and fry body length at termination of experiment) in two natural populations of pike (*Esox lucius*). Grey circles represent Harfjärden, and black diamonds represent Lervik. Figure shows lsmeans ± s.e

Hatching success was in general higher in Harfjärden than in Lervik (Fig. 3a, Additional file 1: Table S1). In addition, there was an effect of the interaction between population and temperature treatment (*F*_2,118_ = 11.08, *P* <  0.0001, Table 1), thus the effect of temperature varied between the populations. Within each population, hatching success was similar in the medium and high temperature treatments; and the interaction effect reflected that Harfjärden had highest hatching success in the low temperature treatment, whilst Lervik had the lowest hatching success in the low temperature treatment (Fig. 3a).

**Table 1:** Effects of population and temperature treatment on offspring performance in *Esox lucius* pike

Trait	Type	num *d.f.*	den *d.f.*	*F*-value	*P*-value
*Hatching success*	*glmer*				
Pop x Treat		2	118	11.08	< 0.0001
*Day degrees until hatching*	*lmer*				
Pop x Treat		2	94.1	4.77	0.008
*Fry survival*	*glmer*				
Pop x Treat		2	110	5.63	0.003
*Fry body length*	*lmer*				
Pop x Treat		2	1701.9	22.85	< 0.0001
*Among family variance in hatching success*	*lm*				
Pop		1	3	4.79	0.12
Treat		1	3	0.10	0.78
Pop x Treat		1	2	1.76	0.32

Comparison of effects of temperature on different offspring performance measures in two populations of *Esox lucius*. Effects of the interaction between population and temperature treatment on hatching success, day degrees until hatching, fry survival, and fry body length (5 days post hatch) are presented. The column ‘Type’ indicates which type of statistical model that was used: generalized linear mixed model (glmer), general linear mixed model (lmer), or general linear model (lm)

### Day degrees until hatching

In general, day degrees until hatching decreased with increasing temperature (Fig. 3b). The number of day degrees until hatching in the medium and high temperature treatments was similar in the two populations (Additional file 1: Table S1). However, in the low temperature treatment, the Harfjärden population had a lower number of day degrees until hatching (Additional file 1: Table S1), and thus developed faster and hatched earlier than the Lervik population (as evidenced by a significant interaction effect between population and temperature treatment, *F*_2,94.1_ = 4.77, *P* = 0.008, Table 1).

### Survival

Fry survival during the first 5 days after hatching was in general high (mean ± s.d. 97.8 ± 7.3%, range 95.5–99.6%; Fig. 3c). The Lervik population had slightly higher survival than the Harfjärden population in both the medium and the high temperature treatments (Additional file 1: Table S1). Conversely, the Harfjärden population had slightly higher survival than the Lervik population in the low temperature treatment (Additional file 1: Table S1). That the effect of temperature differed between the two populations was supported by a significant interaction between temperature treatment and population (*F*_2,110_ = 5.63, *P* = 0.003, Table 1).

### Fry body length

Overall, fry body length increased with increasing temperature (Fig. 3d, Additional file 1: Table S1). However, the results revealed a significant interaction effect between population and temperature treatment (*F*_2,1701.9_ = 22.85, *P* <  0.0001, Table 1). The interaction reflected that fry body length was similar for the two populations in the medium temperature treatment, whereas the Harfjärden population had longer fry than Lervik in both the low and the high temperature treatments (Additional file 1: Table S1).

### Intrapopulation variation in plasticity

**Fig. 4:**
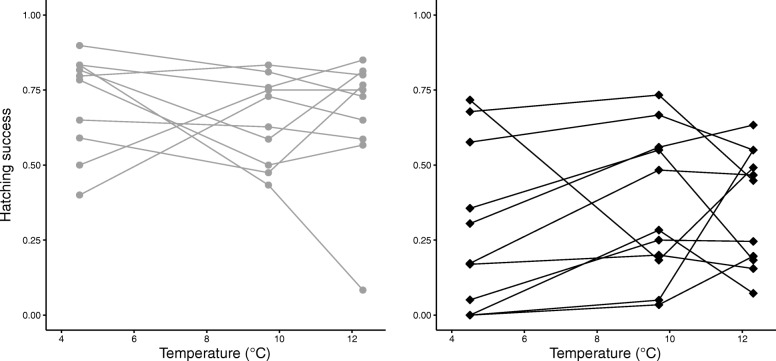
Family-specific responses to temperature. Among family (female/male pair) variation in hatching success across three different temperature (low, medium, high) treatments (G x E) for Harfjärden (grey circles, left panel) and Lervik (black diamonds, right panel). Figure is based on means of the two replicates within each family and temperature treatment. Results for families in the different temperature treatments are connected by lines

The results from the intrapopulation comparisons revealed significant family – environment interaction (G x E) effects on hatching success (Harfjärden: *F*_9,29_ = 6.11, *P* = 0.029, Lervik: *F*_10,20_ = 6.45, *P* = 8.73 × 10^− 5^), indicating that the effects of temperature on hatching success differed among families within both populations (Fig. 4). The comparison of plasticity (among family, within population, variance) in hatching success between the populations revealed that the total variance did not differ between populations (*F*_1,3_ = 4.79, *P* = 0.12, Table 1) or among temperature treatments (*F*_1,3_ = 0.10, *P* = 0.78, Table 1), and that there was no effect of the interaction between population and treatment (*F*_1,2_ = 1.76, *P* = 0.32, Table 1).

## Discussion

To improve projections regarding the effects of global warming it is important to increase the knowledge about how changes in environmental factors, such as temperature, affect different species and populations. This study investigated differentiation and temperature related adaptability of early life history and offspring performance traits in two subpopulations of pike that normally differ in spawning time and in spawning temperatures. Overall, results from the common garden temperature gradient experiment indicated that the early spawning and potentially cold adapted subpopulation performed better in the lowest temperature and that there was genetic differentiation in developmental plasticity between populations. Comparisons among families further indicated the presence of genetic variation for developmental plasticity within both populations.

### Populations were exposed to different temperature regimes

The water temperatures were comparable between the two wetlands (Fig. 2), and the field study confirmed previous observations that the Harfjärden population initiates spawning several weeks earlier than the Lervik population (March 14 and April 4, respectively, in 2017). A likely explanation for this difference in phenology is that the Harfjärden population spawns in a wetland that is located at an altitude above the sea level [40], and where the spring flood occurs early in the spring. The stream connecting the Harfjärden wetland to the Baltic Sea thus experiences a substantial decline in waterflow relatively early in the spring, making migration impossible. In order for the offspring to leave the wetland and migrate to the Baltic Sea before the stream dries out adults in Harfjärden have to initiate spawning early in the spring. The Lervik wetland on the other hand is located at an altitude approximately level with the sea, and the stream connecting the Lervik wetland to the Baltic Sea holds water all spring, thus allowing pikes to spawn later in the spring when the temperature conditions potentially are more favorable. As a consequence of the difference in timing, the temperature at initiation of spawning and incubation of eggs and embryos differed markedly between the two populations, being more than two times higher in Lervik (9.4 ± 1.6 °C) than in Harfjärden (4.3 ± 1.1 °C).

### Effects of temperature were population specific

The results from the experiment revealed that temperature affected all offspring performance measures (hatching success, day degrees until hatching, early fry survival, and fry body length) (Table 1). Hatching success was in general lower in the Lervik population than in the Harfjärden population (Fig. 3a), which is consistent with the results from previous studies conducted both in laboratory and in the field [38–41, 43]. The causes of this difference have not been identified. Berggren et al. [39] showed that hatching success was correlated with differences in suspended material in the spawning grounds causing sedimentation on eggs, and also report that the Lervik females produce relatively small eggs. Based on this it could be hypothesized that the difference between populations in hatching success reflects a life-history trade-off between quality and quantity of eggs [39]. However, further studies would be needed to evaluate this hypothesis.

Fry body length increased with increasing water temperature (Fig. 3d). This is consistent with what one would expect, given that the rate of metabolic processes increase with increasing temperatures, and that temperature is known to be one of the main environmental factors affecting the rate of physiological processes [44, 45].

Consistent with the results from previous studies (e.g. [46–48]), the number of day degrees until hatching decreased with increasing temperature (Fig. 3b). This indicates that the number of day degrees until hatching is not independent of temperature, and that when employed in this simplified manner it is unsuitable as a proxy to estimate development time. The day degree calculations as used for comparative purposes in this and many other studies rely on the implicit assumptions that the relationship linking performance to temperature is linear, isometric, with insignificant performance rates below 0 °C. In reality, thermal performance curves are typically non-linear, subject to evolutionary modifications, and likely to vary among populations [49, 50]. Our present results confirm that the thermal performance curves for embryonic development in pike are non-linear and also show that they differ between the two populations studied here (Fig. 3), pointing to a role of local adaptation.

The overall effect of temperature on offspring performance differed between the two populations (MANOVA, Wilks Lambda, Λ = 0.90, *P* = 0.017), and there were significant interaction effects between population and temperature treatment for all four offspring performance measures (Table 1). This may indicate genetic differentiation in developmental plasticity between populations and that the two populations harbor different local adaptations to temperature during early fry development. Because our data is restricted to the F1-generation, it is possible that non-genetic parental effects influenced the results. However, we produced common garden juveniles by stripping gametes from adults living in a shared environment prior to reproduction [41], and as a result the influence of any environmental parental effect should have been negligible. Nonetheless, differences in maternal condition may have contributed to the overall differences in offspring performance measures between the two populations. Non genetic sire effects comprise another potential source of variation. Recent evidence from an experimental study the European whitefish (*Coregonus lavaretus*) indicates that the thermal environment of the sperm during the period shortly (15 h) before fertilization can impact offspring phenotypes [51]. Despite the caveats regarding the possible contribution of parental effects and transgenerational plasticity, the crossing norms of reaction detected in this study, both at the level of populations and at the level of families within populations, suggest that genetic components were involved.

The adult pike that contributed gametes for the common garden experiment in the present study were sampled over a very narrow time window (3 days) at the onset of the spawning period within each population. A previous study shows that the duration of the spawning migration period lasts for 7 weeks, on average, in the Lervik population [35]. Despite the restricted sampling period, we found that the responses to temperature varied significantly among families in both populations. Yet, it is likely that our results underestimate the adaptability and true genetic variation for developmental plasticity of early life history traits within the study populations. To evaluate this, adult fish that contribute gametes for the common garden experiment need to be sampled on several occasions throughout the spawning period; an approach that would be possible but logistically challenging.

That fish populations with a history of exposure to different thermal conditions during incubation of eggs and embryos in the wild display local adaptations and respond differently to temperature manipulations has previously been demonstrated. Examples include cold water specialist salmonids, such as brown trout (*Salmo trutta*) [52] and grayling (*Thymallus thymallus*) [53–55]. By comparison, pike typically also inhabit warmer waters.

Pike commonly spawn at temperatures in the range of 8 to 12 °C [56–58]. This was the case for the Lervik population. However, the Harfjärden population initiated spawning at a considerably lower temperature.

That neither hatching success nor offspring survival was negatively affected by the low temperature treatment in the Harfjärden population, in combination with the finding that it initiates spawning at a lower temperature than the Lervik population, thus suggests that Harfjärden has evolved population specific adaptations to lower temperatures during early fry development. This is presumably driven by the constraint on spawning time imposed by the restricted time that the wetland is connected to the Baltic Sea. An earlier investigation of the anadromous pike population in Lervik shows that the timing of arrival to the spawning area may vary among years by as much as 3 weeks. Despite this pronounced year-to-year flexibility, the timing of spawning migration differed considerably and in a consistent manner among individuals within the population [35]. Whether this variation has a genetic component remains unknown, but estimates of repeatability indicate that an upper bound of heritability is about 0.25 [35], thus pointing to a potential for an evolutionary response to selection on timing of spawning in pike.

Given that early and late spawning phenotypes coexist within populations [35], and that the offspring produced by early arrivers likely develop in lower temperatures, it can be hypothesized that correlational selection [59, 60] acting on the combination of spawning timing and temperature tolerance has favoured the evolution of genetic covariance and phenotypic integration between these behavioural and physiological traits, as demonstrated for other trait combinations in other organisms [61–63]. This may also have contributed to the differentiation between the two populations studied here. A first step towards evaluating this hypothesis would be to compare the temperature related performance of eggs and embryos produced by gametes collected from adults arriving at the beginning and towards the end of the spawning period.

### Implications for genetic diversity, management and responses to environmental change

Results suggested that early fry development in the Harfjärden population, which is forced to initiate spawning earlier in the spring, is adapted to colder water than the Lervik population. So, what are the implications of these findings for genetic diversity and management?

In the wake of climate change, populations in many environments will be exposed to higher average and more variable and extreme temperatures. There is a variety of ways by which individuals, populations and species can respond to and cope with altered environmental conditions. Common strategies for this are to acclimate [64, 65] or adapt [13, 66, 67] to the novel conditions, or to disperse to habitats with more suitable conditions, which might result in range shifts and expansions [8, 68–70].

In general, the results from the laboratory experiment suggest that the study populations harbor genetic variation and plasticity in temperature tolerance that would make them able to cope with increased temperature, of a magnitude comparable to natural conditions in the wild. However, the conclusion about the temperature tolerance for each population should be interpreted with some caution, because the effects of fixed temperatures (used in the present study) are not necessarily entirely representative of responses to natural fluctuating temperature conditions. Nonetheless, at the population level, the hatching success of the Lervik population was comparable in the medium and high temperatures, and the Harfjärden population only performed slightly better in the lowest temperature than in the medium temperature.

Temperatures during spawning and development of eggs and embryos vary between years. Perhaps counterintuitively, continued global warming may cause additional anadromous pike populations in the Baltic Sea to experience colder - not warmer – waters during spawning, resulting from more variable weather conditions during winter and time constraints caused by drought combined with changes in precipitation and ice cover. Such fluctuating conditions are expected to cause variable selection pressure, favoring different genotypes at different times, which can result in populations consisting of generalists that tolerate a range of conditions, or different specialists [71]. The intrapopulation comparisons revealed that the effects of temperature differed among families (female/male pairs) within both Lervik and Harfjärden (Table 1, Fig. 4), and the total variance in hatching success within and among families did not differ between the populations (Table 1, Fig. 4).

The shape of the reaction norms (Fig. 4) indicate that both populations consist of a mixture of generalists (that perform similarly among the temperature treatments) and specialists. That both populations harbor genetic variation and plasticity in temperature tolerance likely makes them predisposed to cope with increased and more fluctuating temperatures associated with future climate change. However, it is important to keep in mind that the selective regime in the wild comprises not only temperature, and that the responses to altered temperature might be modified by selective pressure imposed by other environmental variables. For example, it has been shown that the effect of salinity is temperature dependent [72]. That the two study locations occasionally experience different salinity regimes [40], might therefore influence the response to altered temperatures. However, to investigate whether and how offspring performance of anadromous pike is affected by the interaction of temperature and salinity would require further studies. It should also be noted that any potential effects of salinity on offspring performance cannot explain the significant interaction effects between population and temperature treatment that were found in the present study.

That the populations seem to harbor sufficient genetic variation and plasticity in temperature tolerance to cope with the projected rise in temperature, in combination with the homing propensity of pike [34], makes it likely that they will adopt the ‘stay and adapt’ strategy. However, the earlier demonstration that time of spawning migration in pike is highly flexible, with individuals fine-tuning migratory timing between years [35], may indicate that they use temporal behavioral adjustments to ensure that their embryos and larvae develop when temperature conditions are favorable. Such phenotypic flexibility may buffer populations against rapid unpredictable environmental changes and potentially prevent the loss of genetic diversity [5, 6].

It is also possible that some individuals will adopt a matching habitat choice behavior [73] and disperse to get to spawn in suitable habitats similar to the thermal environments that they are adapted to. Due to the among-family variation in response to temperature, it is likely that some of the individuals would be more prone to migrate. It can be hypothesized that differential emigration of certain genotypes from a population should decrease intrapopulation genetic variation in the source population. The effect on the genetic variation in the receiving population on the other hand depends on the success of the immigrating individuals. The outcome of such migration is hard to predict, as the response to interpopulation hybridization can differ between different populations [74–78], and in pike even depend on the sex of the migrating individuals [43].

## Conclusions

We found that temperature affected all four offspring performance measures that we investigated, and the effects were population-specific. These differences may be reflective of a population-specific adaptation to low temperature during spawning and early fry development in one population caused by the stream connecting the wetland with the Baltic Sea drying up relatively early, forcing the individuals to spawn early in the spring. Our findings confirm that water temperature is one of many environmental variables that impose selective pressures on and drive local adaptations in pike. We also found that the effects of temperature differed among families within both populations (Fig. 4). Such intrapopulation genetic variation for developmental plasticity offers buffering capacity and adaptive potential, and is thus key to resilience and long-term survival in the face of global warming and environmental change.

## Methods

### Study populations

The two anadromous populations of pike used in this study spawn in wetlands in the Southeast of Sweden. One of the locations, Lerviksbäcken (henceforth Lervik, N 57° 04.414′; E 16° 31.246′), is located on the East coast of the Swedish mainland and the other location, Harfjärden (N 56° 49.063′; E 16° 48.673′), is located on the East coast of the island of Öland (Fig. 1). The two populations are separated by approximately 120 km (shortest waterway distance), and show a strong neutral genetic differentiation, as evidenced by results from previous analysis of data on microsatellite markers (pairwise FST = 0.226, [22, 37]). The localities also differ in altitude, with the spawning area in Lervik being located ca 25 cm above mean sea level, whereas that in Harfjärden is located > 100 cm above the mean sea level [40]. Due to this, the spawning area in Lervik is occasionally influenced by backflow of brackish water from the Baltic Sea [40].

Observations in the spawning areas during previous years demonstrate that the two populations differ considerably in their time of spawning. The timing of spawning migration in the Lervik population has been studied in great detail [35]. Data from a 6-year mark-recapture study, including > 2000 individuals, showed that the time and duration of the period within which individuals arrived to the breeding site varied across years. Over the period 2006–2011, the first day of arrival to the Lervik spawning area varied between March 20 and April 6, whereas the final day of arrival varied between May 6 and May 25 [35]. By comparison, the timing of spawning migration in the Harfjärden population has not been studied as intensively. However, previous unpublished observations and results of the present study showed that over the period 2016–2018, the first day of arrival to the Harfjärden spawning area varied between February 21 and March 14, and demonstrate that the Harfjärden population generally initiate spawning a few weeks prior to the Lervik population (see Results). This difference is likely explained by the relatively high altitude of the Harfjärden wetland, in combination with an early spring flood. These factors cause the stream leading up to the wetland to usually dry out relatively early compared with other spawning grounds used by anadromous pike in this area. This restricts the window of opportunity for spawning for the Harfjärden population, forcing them to spawn earlier in the spring.

### Field observations

To determine when spawning started in the spring of 2017, observations in the spawning locations were carried out daily from March 1 in Harfjärden and March 30 in Lervik (initialized before the onset of spawning). At the time of the first observation in each location, two temperature loggers (HOBO Pendant) were placed in the water to track daily and seasonal changes in water temperature. The water temperature was recorded every 10 min until April 15 (throughout the sampling period, and 30 and 9 days from start of the spawning for Harfjärden and Lervik, respectively).

### Sampling

Pikes were captured using fyke nets (with the opening of the fyke-nets directed toward the sea) placed directly downstream of the wetland outlet (Lervik), or just inside the outlet from the wetland (Harfjärden), and sampling in each location was started as soon as ripe individuals arrived to the spawning location (March 14 in Harfjärden, and April 4 in Lervik). Because the two populations differed in spawning time (see ‘Field observations and temperature measurements’ in results), sampling of individuals had to be carried out during two periods (Harfjärden March 14–16 and Lervik April 4–6, 2017). Out of the ripe individuals, 10 males and 10 females from each location were stripped for gametes (by gently massaging the abdomen and collecting eggs in 50 ml Falcon tubes and milt in 2 ml Eppendorf tubes), measured for body length (mean ± sd; Harfjärden_females_ = 66,4 ± 5,8 cm, Harfjärden_males_ = 54,4 ± 6,9 cm, Lervik_females_ = 76,8 ± 10,8 cm, Lervik_males_ = 58,7 ± 6,5 cm), and then immediately released back into the water. To ensure high quality eggs, only females with eggs without visual traces of blood were used. To ensure that the eggs had not been in contact with water (to avoid premature opening of the micropyle [57, 79]), the first batch of eggs from each female was discarded [39, 40, 43]. The tubes with gametes were immediately placed on ice in cooler boxes, and continuously kept on ice during transportation to the Kalmarsound Laboratory of Linnaeus University in Kalmar, Sweden, until artificial fertilization for the temperature tolerance experiment was carried out.

### Common garden temperature tolerance experiment

To evaluate local adaptation and compare between and within population genetic variation for temperature related developmental plasticity of early life history traits, we performed a common garden experiment. For this temperature tolerance experiment, 10 families (female/male pairs) were created for each population by randomly assigning each male to a female, and used in an artificial fertilization, split-brood temperature gradient experiment. Each family was tested in three different temperatures (4.5 °C, 9.7 °C and 12.3 °C), and contributed with two replicates to each treatment, resulting in 120 experimental units (2 populations, 10 families per population, 3 temperature treatments per family, and duplicates per family and treatment). The temperatures were chosen to include two treatments corresponding to the natural spawning temperatures for the study populations (estimated based on observations from previous years – and confirmed by the results in the present study), and one treatment slightly exceeding the natural spawning temperature at initiation of spawning for both of the study populations.

Artificial fertilization was carried out using a somewhat modified version of the method previously used [39, 40, 43]. In short, 30 eggs from each female were placed in a small glass bowl (50 mm ∅), and an excess of milt (approximately 200 μl) from one male were added to the eggs. Following addition of milt, water (approximately an equal volume to the eggs) was also added. The eggs and milt were mixed gently, and the eggs were then let to rest for 2 min before excess milt was removed by rinsing the eggs three times with water. Immediately after rinsing, the eggs were transferred to a separate container and placed in a water bath with circulating water of treatment specific temperature. The containers were randomly distributed within the treatments (water baths) to minimize systematic errors due to differences in light, or potential within-treatment differences in temperature. The eggs, and subsequently hatched fry, were continuously kept in their natal container in the water bath throughout the experiment.

Each container consisted of two 800 ml plastic cups placed in each other, with the bottom of the inner bin replaced with a plastic net (mesh size 1.5 × 1.5 mm) [40, 43]. This allowed for water exchanges even during the incubation time when eggs are sensitive to movements. Partial water exchanges were done every fifth day by addition of water (250 ml) to each container. To minimize effects of non-treatment specific temperatures, water with treatment specific temperature was always used in both the artificial fertilization procedure and for water exchanges.

To get accurate measurements of the water temperature within the experimental units, two temperature loggers (HOBO Pendant) per treatment were placed in separate unused (without eggs) experimental containers and randomly placed in the water baths along with the experimental units. The loggers recorded the temperature every 10 min, and the containers with temperature loggers were kept, as the real experimental units, in the water baths throughout the entire experimental period.

The eggs in each unit were inspected daily, and dead eggs removed with a plastic pipette. To monitor the progress of fry development and hatching, photographs of each unit, showing eggs and larvae, were taken every morning (~ 9 am) throughout the experiment, and during the time of hatching an additional photo was taken in the evening (~ 8 pm) (using a Panasonic DMC-TZ5). Each unit was terminated 5 days post hatch (defined as the time when 50% of the remaining eggs had hatched), by euthanizing the remaining fry with an overdose of benzocaine (immersion in 250 mg/L). At termination an additional photo was then taken of the fry against the net bottom for subsequent length measures [40, 43].

### Data collection

Data collection included water temperature measurements from the laboratory and the field, and quantification of four offspring performance measures: *i*) proportion of eggs that successfully hatched; *ii*) day degrees until hatching; *iii*) survival of hatched fry during 5 days; and *iv*) fry body length 5 days post hatch.

Water temperatures were directly obtained from the temperature loggers placed in the wetlands and in the laboratory (using software HOBOware). Data on offspring performance measures was collected by analyzing the picture series taken during the experiment, in combination with the data from the temperature measurements in the laboratory. Hatching success and survival were directly extracted from the picture series by visual inspection and counting of eggs and fry [40, 43]. Data on day degrees until hatching was obtained by multiplying number of days until hatching for each fry with the mean temperature in the treatment during the incubation time. Fry body length was measured from the pictures of fry against the net bottom (utilizing the net as a ruler, and using software ImageJ) [40, 43].

### Statistical analyses

First, we tested if parental lengths differed between the two populations. To this end we ran a separate linear model for each of the sexes with parental body length as response variable and population as explanatory variable (using the Stats package version 3.5.0 in RStudio 2 v1.1.383 [80], with R v.3.2.2 [81]). This showed that body length differed between the populations for both males and females (*P*_*males*_ <  0.0001, *P*_*females*_ <  0.0001). To determine whether body length should be included as a covariate in the subsequent analyses, we then tested whether offspring performance was associated with parental lengths. To test this we ran additional separate linear models for hatching success, survival, day degrees until hatching and fry body length for each of the sexes. Parental length was treated as a covariate, temperature treatment as fixed categorical factor, and the interaction between parental length and temperature treatment was also included. As none of the tests showed significant interaction effects, we re-ran the analyses with the same dependent and fixed factors excluding the interaction term. None of the offspring performance measures (hatching success, fry survival, or fry body length) were significantly associated with either maternal or paternal length (hatching success: *P*_*males*_ = 0.45, *P*_*females*_ = 0.79; survival: *P*_*males*_ = 0.52, *P*_*females*_ = 0.85; day degrees: *P*_*males*_ = 0.13, *P*_*females*_ = 0.17; fry body length: *P*_*males*_ = 0.68, *P*_*females*_ = 0.89). Parental lengths were therefore not included as explanatory variables in the following analyses.

To test whether population, temperature treatment, or the interaction between population and temperature treatment had an overall effect on offspring performance we performed a MANOVA including all four offspring performance measures (hatching success, day degrees until hatching, fry survival, and fry body length) as response variables (Stats package version 3.5.0). Population and temperature treatment were treated as fixed categorical factors, and the interaction between population and treatment was included. As the test revealed a significant interaction effect (Λ = 0.90, *P* = 0.017), additional separate analyses were carried out for each of the four offspring performance measures to disentangle whether and how each of them differed between populations, was affected by temperature, and whether the responses to temperature varied between and within populations. Different types of models were selected depending on the distribution of the response variable. Data on hatching success and fry survival, with a binary response variable, was analyzed with generalized linear mixed models with a binomial fit and a logit-link function using the lme4 package (version 1.1–15, glmer function). Data on day degrees until hatching and fry body length, with normal response distributions, were analyzed with general linear mixed models (lmer function). For all interpopulation comparisons, population and temperature treatment were treated as fixed categoric factors, and family (female/male pair) and replicate (nested in family) were treated as random factors. Results and conclusions for day degrees and fry length remained qualitatively unchanged when modelled with gamma rather than normal distributions.

To test whether and how the effects of temperature differed among families within the populations, we performed separate family – environment (G x E) interaction analyses for each of the populations. To avoid using biased estimates resulting from differences in hatching success, this was only tested for data on hatching success. The data was analyzed with generalized linear mixed models using the lme4 package (with the glmer function), with temperature treatment and family (female/male pair) treated as a fixed categorical factors, and replicate as a random factor. To test if the variance in hatching success among families differed between populations or temperature treatment, or whether it was affected by the interaction between population and treatment, we calculated the variance in hatching success for each treatment within each population, and tested it with a general linear model (lm function in R).

For all tests (both inter- and intra-population comparisons) statistical significance of fixed and interaction effects were assessed using the Type III test of fixed effects, and hypotheses were tested against an α–level of 0.05. If there was no significant interaction effect, the interaction term was excluded and a test of main effects was run. Degrees of freedom was approximated with Satterthwaite’s method.

Our analyses uncovered significant effects of the interaction between population and temperature treatment on all four response variables (see Results). We therefore also performed separate analyses of subsets of the data. Results from tests for differences between populations within each temperature treatment and for differences between temperature treatments within each population are reported in Additional file 1: Table S1.

## Additional file

Additional file 1: **Table S1** Effects of population (Pop) for each temperature treatment (low: 4.5 °C, medium: 9.7 °C and high: 12.3 °C) and effects of temperature treatment (Treat) for each population (Harfjärden and Lervik) on hatching success, day degrees until hatching, fry survival, and fry length. The column ‘Type’ indicates which type of model that was used: generalized linear mixed model (glmer), general linear mixed model (lmer), or general linear model (lm). (DOCX 42 kb)

## Data Availability

The datasets supporting the conclusions of this article are publicly available in the Dryad Digital repository (10.5061/dryad.2c6951v) [82]. Data on hatching success and survival in performance.xlsx, data on day degrees until hatching in daydegrees.xlsx, and data on fry body length in length.xlsx.

## Abbreviation

G x E: Gene by environment interaction
