# Supplementary material for: Adaptations of early development to local spawning temperature in anadromous populations of pike (Esox lucius)
Source: BMC Evol Biol. 2019 Jul 22;19:148. doi: 10.1186/s12862-019-1475-3 (PMC6647320; doi:10.1186/s12862-019-1475-3)
Supplement: Supplementary file 1 — Table S1 Effects of population (Pop) for each temperature treatment (low: 4.5 °C, medium: 9.7 °C and high: 12.3 °C) and effects of temperature treatment (Treat) for each population (Harfjärden and Lervik) on hatching success, day degrees until hatching, fry survival, and fry length. The column ‘Type’ indicates which type of model that was used: generalized linear mixed model (glmer), general linear mixed model (lmer), or general linear model (lm). (DOCX 42 kb) [file 12862_2019_1475_MOESM1_ESM.docx]

**Additional file 1**

**Table S1.** Effects of population (Pop) for each temperature treatment (low: 4.5 °C, medium: 9.7 °C and high: 12.3 °C) and effects of temperature treatment (Treat) for each population (Harfjärden and Lervik) on hatching success, day degrees until hatching, fry survival, and fry length. The column ‘Type’ indicates which type of model that was used: generalized linear mixed model (glmer), general linear mixed model (lmer), or general linear model (lm).

| **Trait** | **Type** | **num *d.f.*** | **den *d.f.*** | ***F*-value** | ***P*-value** |
| --- | --- | --- | --- | --- | --- |
| *Hatching success* | *glmer* |  |  |  |  |
| Pop (low) |  | 1 | 37 | 16.15 | < 0.0001 |
| Pop (medium) |  | 1 | 37 | 10.68 | 0.0011 |
| Pop (high) |  | 1 | 37 | 9.74 | 0.0018 |
| Treat (Har) |  | 2 | 55 | 3.03 | 0.049 |
| Treat (Ler) |  | 2 | 61 | 8.84 | 0.0001 |
|  |  |  |  |  |  |
| *Day degrees until hatching* | *lmer* |  |  |  |  |
| Pop (low) |  | 1 | 16.4 | 5.05 | 0.02 |
| Pop (medium) |  | 1 | 19.1 | 1.05 | 0.31 |
| Pop (high) |  | 1 | 19.0 | 0.02 | 0.88 |
| Treat (Har) |  | 2 | 47.3 | 592.06 | < 0.0001 |
| Treat (Ler) |  | 2 | 47.1 | 301.01 | < 0.0001 |
|  |  |  |  |  |  |
| *Fry survival* | *glmer* |  |  |  |  |
| Pop (low) |  | 1 | 30 | 0.34 | 0.42 |
| Pop (medium) |  | 1 | 37 | 0.08 | 0.77 |
| Pop (high) |  | 1 | 35 | 0.12 | 0.45 |
| Treat (Har) |  | 2 | 54 | 7.34 | 0.0005 |
| Treat (Ler) |  | 2 | 53 | 7.93 | 0.0002 |
|  |  |  |  |  |  |
| *Fry length* | *lmer* |  |  |  |  |
| Pop (low) |  | 1 | 34.0 | 10.58 | 0.003 |
| Pop (medium) |  | 1 | 17.8 | 0.30 | 0.59 |
| Pop (high) |  | 1 | 18.6 | 4.19 | 0.06 |
| Treat (Har) |  | 2 | 1076.3 | 1432.80 | < 0.0001 |
| Treat (Ler) |  | 2 | 613.8 | 822.71 | < 0.0001 |
